# The epidemiology of severe sepsis in England, Wales and Northern Ireland, 1996 to 2004: secondary analysis of a high quality clinical database, the ICNARC Case Mix Programme Database

**DOI:** 10.1186/cc4854

**Published:** 2006-03-10

**Authors:** David A Harrison, Catherine A Welch, Jane M Eddleston

**Affiliations:** 1 Intensive Care National Audit & Research Centre (ICNARC), Tavistock House, Tavistock Square, London WC1H 9HR, UK; 2 Manchester Royal Infirmary, Oxford Road, Manchester M13 9WL, UK

## Abstract

**Introduction:**

To evaluate the impact of recent evidence-based treatments for severe sepsis in routine clinical care requires an understanding of the underlying epidemiology, particularly with regard to trends over time. We interrogated a high quality clinical database to examine trends in the incidence and mortality of severe sepsis over a nine-year period.

**Methods:**

Admissions with severe sepsis occurring at any time within 24 hours of admission to critical care were identified to an established methodology using raw physiological data from the Intensive Care National Audit & Research Centre (ICNARC) Case Mix Programme Database, containing data from 343,860 admissions to 172 adult, general critical care units in England, Wales and Northern Ireland between December 1995 and January 2005. Generalised linear models were used to assess changes in the incidence, case mix, outcomes and activity of these admissions.

**Results:**

In total, 92,672 admissions (27.0%) were identified as having severe sepsis in the first 24 hours following admission. The percentage of admissions with severe sepsis during the first 24 hours rose from 23.5% in 1996 to 28.7% in 2004. This represents an increase from an estimated 18,500 to 31,000 admissions to all 240 adult, general critical care units in England, Wales and Northern Ireland. Hospital mortality for admissions with severe sepsis decreased from 48.3% in 1996 to 44.7% in 2004, but the total number of deaths increased from an estimated 9,000 to 14,000. The treated incidence of severe sepsis per 100,000 population rose from 46 in 1996 to 66 in 2003, with the associated number of hospital deaths per 100,000 population rising from 23 to 30.

**Conclusion:**

The population incidence of critical care admission with severe sepsis during the first 24 hours and associated hospital deaths are increasing. These baseline data provide essential information to those wishing to evaluate the introduction of the Surviving Sepsis Campaign care bundles in UK hospitals.

## Introduction

Severe sepsis is a syndrome characterised by systemic inflammation, coagulopathy and acute organ dysfunction in response to infection [1]. The published mortality associated with the disease has reduced slightly in the past 10 to 15 years, almost certainly a reflection of improved supportive clinical care, but still remains high (30% to 50%) [2]. This reduction is evident from comparative outcomes in placebo groups of large randomised studies in severe sepsis [3-6]. The challenge is to achieve outcomes for patients that are consistent with the treatment limbs of these recent studies. Recent treatment modalities that have established their efficacy in patients with severe sepsis include drotrecogin alfa (activated) [5] and early goal-directed therapy [7]. The widespread adoption of such evidence-based practice into clinical care has been disappointingly slow, despite the quantifiable benefits of a 6.1% absolute reduction in 28-day mortality with drotrecogin alfa (activated) [5] and a 16% absolute reduction in hospital mortality with early goal-directed therapy [7].

The Surviving Sepsis Campaign was developed in an attempt to address the clinical inertia in the adoption of such evidence-based strategies [8]. The campaign has worldwide support from professional societies and has gained consensus on the management of patients with severe sepsis. The guidelines have subsequently been deployed in two bundles, with the components in each bundle sharing a common relationship in time.

Clinical experience to date would suggest that the inclusion of evidence-based strategies into bundles, which facilitate a drive to change by the reduction of omissions of clinical care, will be the key to effective change in practice [9]. Quantifying the full impact of these changes will require a longitudinal understanding of the underlying epidemiology of severe sepsis in the population. It is important to know not only baseline data, such as the incidence of severe sepsis and associated mortality, but also trends in these data over time, so that any change can be evaluated relative to these trends. Many recent studies have explored the epidemiology of severe sepsis in different populations [10]; however, most of these studies had a short time frame and were unable to describe changes over time.

We present data from an analysis of a database arising from a national audit of patient outcomes from critical care units in England, Wales and Northern Ireland. Data from this database have previously been used to establish baseline epidemiology for severe sepsis in the UK [11,12]. When these analyses were performed, however, only four years' data were available, limiting the usefulness of comparisons over time. The increasing amount of data from this ongoing audit means that a more complete analysis of time trends is now possible. In this article, we present an analysis of the changes in the epidemiology of severe sepsis presenting within 24 hours of admission to critical care in England, Wales and Northern Ireland. The data cover the nine-year period from 1996 to 2004.

These results were presented at the Surviving Sepsis Campaign: Launch of the Care Bundles in England, Manchester Royal Infirmary, 13 June 2005.

## Materials and methods

### Case Mix Programme Database

The Case Mix Programme Database is a high quality clinical database containing data on demographics, case mix, outcome and activity for admissions to adult, general critical care units participating in the Case Mix Programme. The Programme provides a national comparative audit of critical care for England, Wales and Northern Ireland and is co-ordinated by the Intensive Care National Audit & Research Centre (ICNARC) [13]. Patient data are abstracted by trained data collectors according to precise rules and definitions, and are subject to both local and central validation before being pooled into the database. The database includes physiology data from the first 24 hours following admission to the critical care unit and reason for admission to the critical care unit. In total, data for 343,860 admissions to 172 critical care units between December 1995 and January 2005 were available for analysis. The critical care units included intensive care units (ICUs) and combined ICU/high dependency units (HDUs), but not stand-alone HDUs.

### Selection of cases

Admissions with severe sepsis during the first 24 hours in the critical care unit were identified using physiological criteria derived from those used in the Protein C Worldwide Evaluation in Severe Sepsis (PROWESS) study of drotrecogin alfa (activated), as described previously [12]. Briefly, severe sepsis was defined as evidence of infection plus three or more systemic inflammatory response syndrome (SIRS) criteria [1] and at least one organ dysfunction (cardiovascular, respiratory, renal, haematological or metabolic) during the 24-hour period. This diagnosis of sepsis was based on raw physiological data.

### Analyses

The total number of admissions with severe sepsis within 24 hours of admission to critical care and percentage of all admissions was calculated for each year from 1996 to 2004. For admissions with severe sepsis, the case mix, outcome and activity were described for each year.

Case mix was measured by age, sex, severe comorbidities, surgical status (admissions direct from theatre following elective or emergency surgery, and non-surgical admissions), pre-admission critical care (transfers from another ICU or HDU, and those managed on the ward by the critical care team prior to admission to the critical care unit), reason for admission to the critical care unit, Acute Physiology and Chronic Health Evaluation (APACHE) II score [14], number of organ dysfunctions, and summaries of individual physiological variables. Outcome was measured by the mortality in the critical care unit, transfers out of the critical care unit for ongoing care in another ICU or HDU, and the mortality at ultimate discharge from an acute hospital, both overall and by surgical status. Activity was measured by the length of stay in the critical care unit (by survival status), the percentage of critical care unit bed-days occupied by admissions with severe sepsis, and the total length of stay in hospital (by survival status).

Trends over time were tested using generalised linear models: logistic regression for binary variables (admissions with severe sepsis, sex, mortality); linear regression for changes in means (age, APACHE II score); ordered logistic regression for ordered categorical data (number of organ dysfunctions); and multinomial logistic regression for unordered categorical data (surgical status, pre-admission critical care, critical care transfers). Median regression was used to test for changes in median lengths of stay. All generalised linear models were fitted with robust (Huber-White) standard errors adjusted for clustering on critical care unit [15].

### Population incidence

The projected total numbers of admissions aged 16 years or over to adult, general critical care units in England, Wales and Northern Ireland for each year were estimated by extrapolating the number of observed admissions to 240 critical care units identified from the Directory of Critical Care [16]. Confidence intervals were estimated by bootstrapping [17]. The projected numbers of admissions were converted to population (treated) incidences by dividing by population estimates obtained from National Statistics [18]. Population estimates were not available for the year 2004.

All analyses were performed using Stata 8.2 (StataCorp LP, College Station, TX, USA). *P *< 0.05 was considered to represent a statistically significant result.

## Results

**Figure 1:**
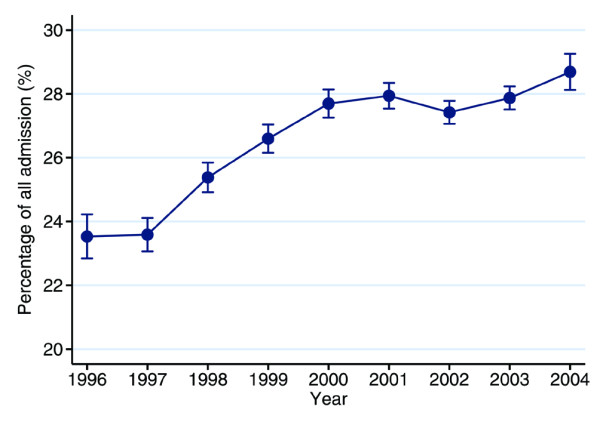
Percentage of admissions with severe sepsis during the first 24 hours following admission to critical care, 1996 to 2004. Percentage for each year with 95% confidence interval.

Of the 343,860 admissions between December 1995 and January 2005, 92,672 (27.0%) were identified as having severe sepsis during the first 24 hours following admission to the critical care unit. The breakdown of the data by year is shown in Table 1. Fifty-five admissions from December 1995 and 78 admissions from January 2005 were included in the totals, but excluded from all analyses by year. The proportion of admissions with severe sepsis during the first 24 hours increased from 23.5% in 1996 to 28.7% in 2004 (*P *= 0.004; Figure 1).

**Figure 2:**
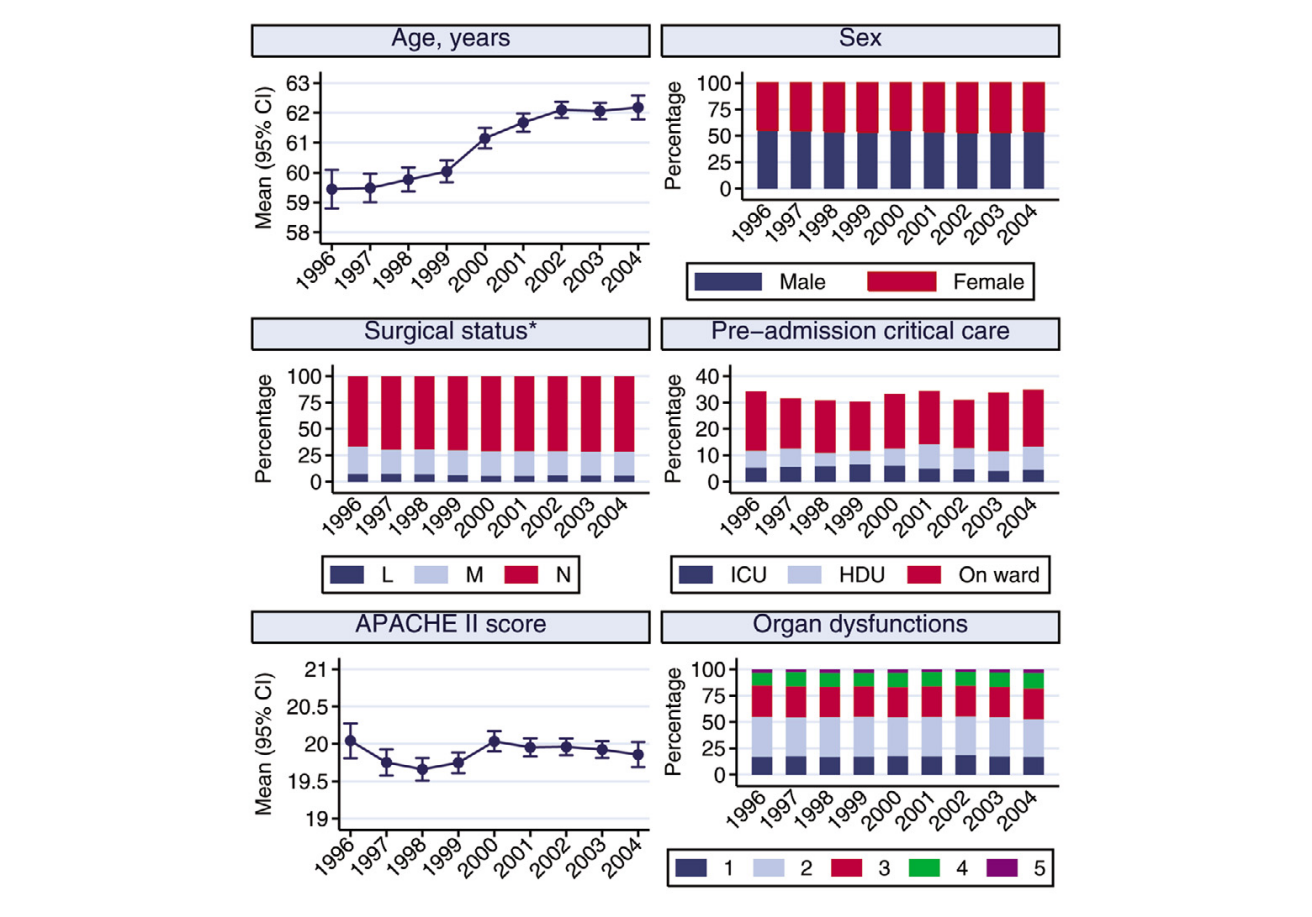
Changes in case mix for admissions with severe sepsis during the first 24 hours following admission to critical care, 1996 to 2004. *Surgical status: L, elective; M, emergency; N, non-surgical. CI, confidence interval; HDU, high dependency unit; ICU, intensive care unit.

Figure 2 shows changes in the case mix of admissions with severe sepsis during the first 24 hours following admission to the critical care unit between 1996 and 2004. The mean age of admissions rose from 59.5 years in 1996 to 62.2 years in 2004 (*P *< 0.001). The sex distribution remained approximately constant (*P *= 0.062), with around 54% of admissions being male. A decreasing proportion of patients were admitted following elective surgery (*P *= 0.046 relative to non-surgical admissions), but there was no change in the relative proportions of emergency surgical and non-surgical admissions (*P *= 0.22). There was a slight decrease in the proportion of admissions with severe sepsis transferred in from another ICU (*P *= 0.003) and a corresponding increase in the proportion stepped-up from an HDU (*P *= 0.036); however, there was no change in the proportion managed on the ward by the critical care team prior to admission (*P *= 0.62). There was no change in the mean APACHE II score (*P *= 0.56) or the number of organ dysfunctions (*P *= 0.84). Overall, 23% of admissions had an APACHE II score of 25 or more (indicating a high risk of death [8]). However, 83% of admissions had multiple organ failure (two or more organ dysfunctions).

Changes in the individual physiological measurements contributing to the SIRS criteria, markers of organ dysfunction, and laboratory measurements are shown in Tables 2, 3 and 4, respectively. There have been some slight trends in the physiological measurements over time, although not sufficient to influence a summary score such as APACHE II. The proportion of severe sepsis admissions that were mechanically ventilated during the first 24 hours in ICU has also decreased from 81% in 1996 to 73% in 2004. Changes in severe comorbidities are shown in Table 5. There has been a decrease in the proportion of severe sepsis admissions with severe respiratory or cardiovascular comorbidities and a slight increase in liver comorbidities.

The most commonly recorded primary reasons for admission to ICU were pneumonia (22.6%), septicaemia/septic shock (11.8%), bowel perforation or rupture (10.5%), exacerbation of chronic obstructive pulmonary disease/asthma (5.4%), meningitis (2.1%) and pancreatitis (1.9%). There was little change in these reasons for admission over time.

**Figure 3:**
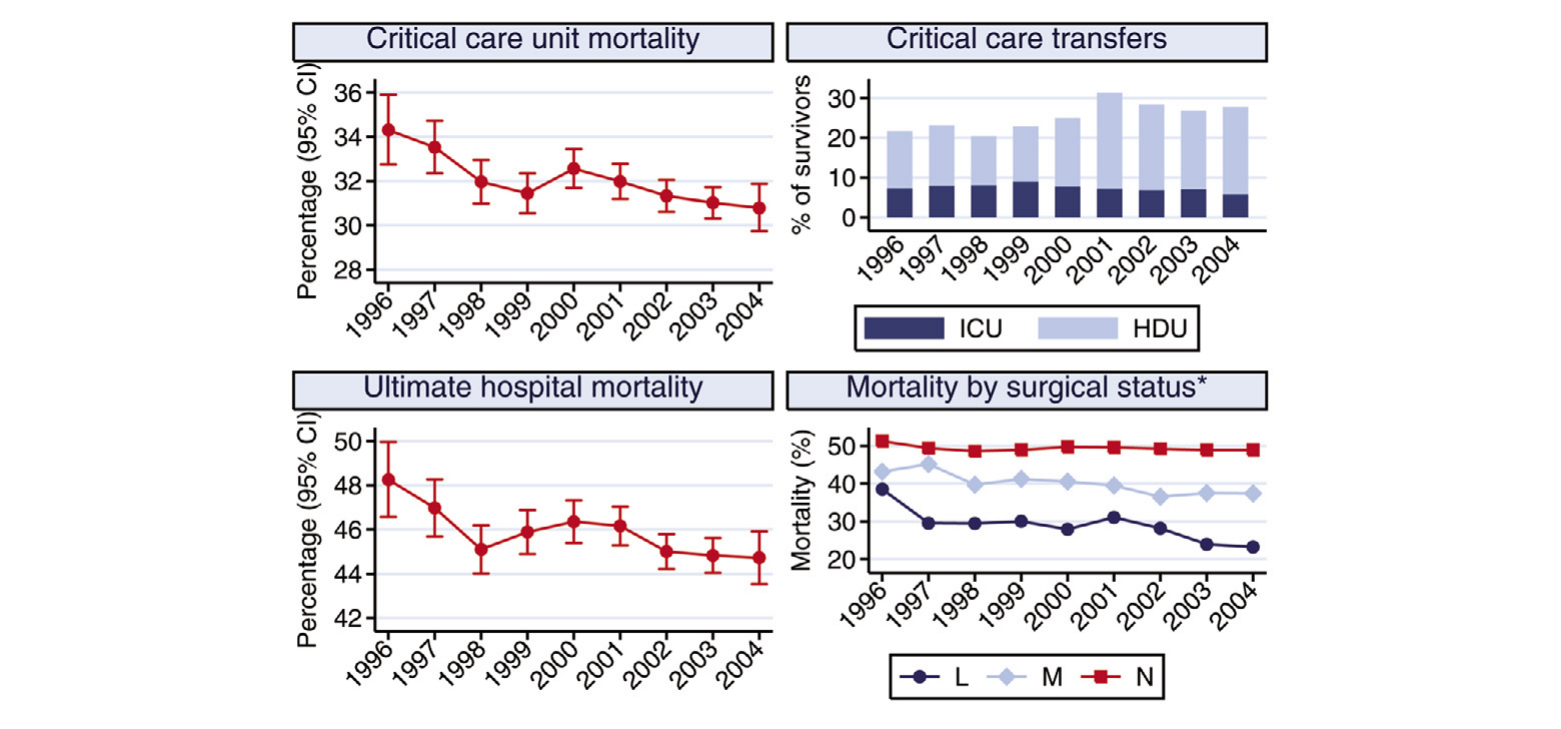
Changes in outcomes for admissions with severe sepsis during the first 24 hours following admission to critical care, 1996 to 2004. *Surgical status: L, elective; M, emergency; N, non-surgical. CI, confidence interval; HDU, high dependency unit; ICU, intensive care unit.

Figure 3 shows changes in unit and hospital outcomes over time. Mortality in the critical care unit decreased from 34.3% in 1996 to 30.8% in 2004 (*P *= 0.013). The proportion transferred to another ICU remained approximately constant at around 7% (*P *= 0.29); however, the proportion transferred to an HDU increased (*P *= 0.009). Hospital mortality also decreased from 48.3% in 1996 to 44.7% in 2004 (*P *= 0.042). The decrease in hospital mortality was more pronounced in surgical admissions (both elective and emergency) than non-surgical admissions.

**Figure 4:**
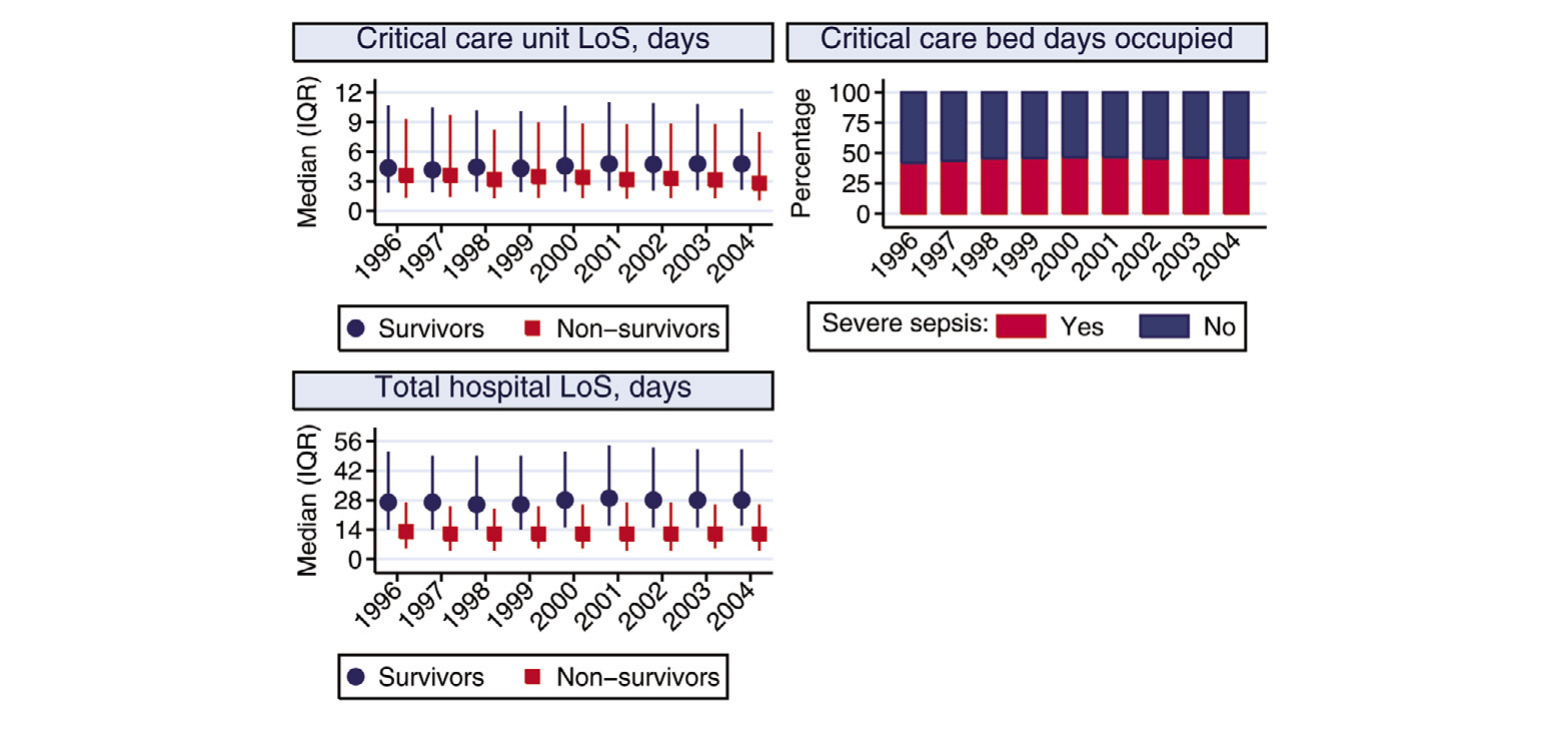
Changes in activity for admissions with severe sepsis during the first 24 hours following admission to critical care, 1996 to 2004. IQR, interquartile range; LoS, length of stay.

Figure 4 shows changes in activity over time. Length of stay in the critical care unit was longer for survivors than non-survivors (median 4.6 versus 3.2 days). This gap widened during the course of the study period as the median length of stay for survivors increased and the median length of stay for non-survivors decreased (both *P *< 0.001). Length of stay for admissions with severe sepsis was long compared to other admissions, indicated by the 27% of admissions that had severe sepsis utilising 46% of all critical care unit bed-days in these units. Median total length of stay in hospital for survivors was four weeks, with a small but statistically significant increase during the study period (*P *< 0.001). There was no change in the median hospital length of stay for non-survivors, which remained constant at 12 days (*P *= 1.0).

**Figure 5:**
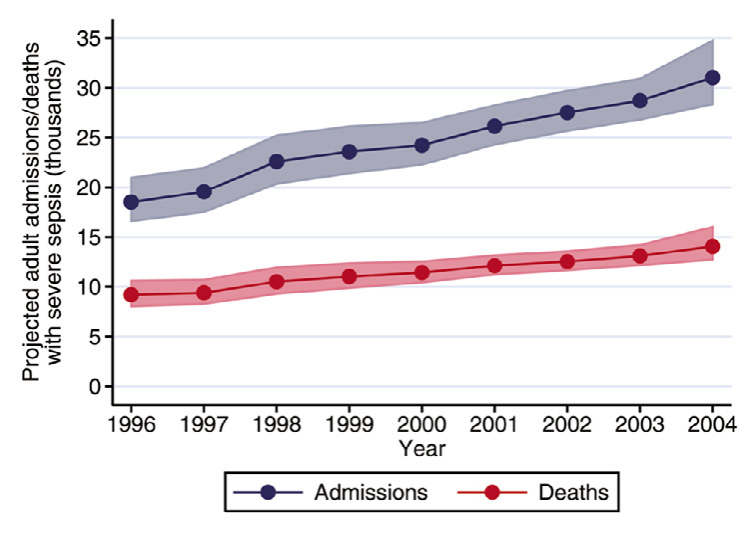
Projected total numbers of admissions to 240 adult, general critical care units with severe sepsis during the first 24 hours following admission to critical care and associated hospital deaths. Projected total for each year with 95% confidence interval. Admissions aged < 16 years and readmissions within the same hospital stay excluded.

**Figure 6:**
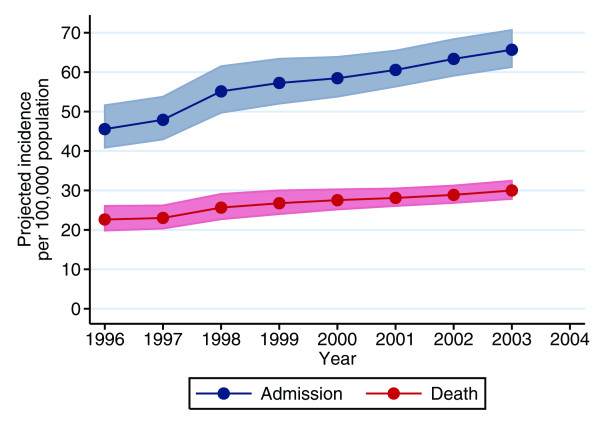
Population incidence of severe sepsis during the first 24 hours following admission to critical care and associated hospital deaths. Incidence per 100,000 population aged 16 years and over for each year with 95% confidence interval.

The projected total numbers of adults (age 16 years or over) admitted to 240 general critical care units in England, Wales and Northern Ireland with severe sepsis during the first 24 hours following admission to the unit, and the corresponding numbers of hospital deaths, are shown in Figure 5. The number of critical care admissions with severe sepsis has risen steeply from 18,500 in 1996 to 31,000 in 2004, and, despite falling mortality, the total projected deaths has risen from 9,000 in 1996 to 14,000 in 2004. These figures are presented as population incidences in Figure 6. The incidence of critical care admission with severe sepsis during the first 24 hours has risen from 46 per 100,000 adult population in 1996 to 66 per 100,000 adult population in 2003. Associated hospital deaths rose from 23 per 100,000 adult population in 1996 to 30 per 100,000 adult population in 2003.

## Discussion

### Key findings

The proportion of critical care admissions with severe sepsis during the first 24 hours rose from 23.5% in 1996 to 28.7% in 2004. Extrapolating to all adult, general critical care units in England, Wales and Northern Ireland, we estimate that there were 31,000 admissions with severe sepsis in 2004 (an increase from 18,500 admissions in 1996). Mortality for admissions with severe sepsis has decreased despite measures of severity of illness (APACHE II score, organ dysfunctions) remaining constant, but the total number of deaths has increased from 9,000 in 1996 to 14,000 in 2004. In population terms, the treated incidence of severe sepsis per 100,000 population rose from 46 in 1996 to 66 in 2003, with the associated number of hospital deaths per 100,000 population rising from 23 to 30.

### Strengths and weaknesses of this study

The main strength of this study is the use of a large clinical database with detailed physiological data for the first 24 hours in ICU. By comparison with studies based on hospital discharge data coded with, for example, International Classification of Diseases (ICD) codes [19-21], we were much better able to match the consensus definitions of severe sepsis. As the definitions were applied to raw physiological data, this removed the potential to bias evaluations of trend over time by changes in the interpretation or application of the definitions by individuals. However, the data were not collected for the primary purpose of identifying severe sepsis and some of the definitions used in the PROWESS study could only be approximated. It was also not possible to identify severe sepsis occurring later than 24 hours after admission to the critical care unit. The long time period (nine years) allowed comparisons over time that have not been possible in previous studies of critical care.

A number of other factors may also have affected the results. The number of units that contributed to the database varied over time. Some closed, merged or left the programme, while new units would join the programme and begin to contribute to the dataset. Hence, variability in the quality of care in different units may also contribute, to some extent, to the results. Severity of disease may also have changed over time, despite little change in APACHE II scores, as indicated by the decreasing proportion of patients receiving mechanical ventilation. Finally, although sepsis was diagnosed using raw data, the criteria used by physicians to admit patients into their ICU may have changed over time. For example, improved recognition of sepsis may have led to earlier admission of patients with sepsis, and this may influence the outcome of the admitted patients.

### Comparison with other studies

By comparison with other countries, the percentage of critical care admissions with severe sepsis in our study is very high. Other studies have reported a range from 7.9% of admissions in the Slovak Republic [22] and 8.4% in France [23] to 14.8% in an international cohort [24] and 17.4% in Brazil [25]. This difference could be due to poor matching of the PROWESS definitions identifying too many patients as having severe sepsis. However, this seems unlikely, as the population incidence is similar to that reported in other studies (47/100,000 in Norway [20], 54/100,000 in the Netherlands [26], 77/100,000 in Australia and New Zealand [27], and 75 to 119/100,000 in the USA [21]). The difference may, therefore, reflect a lower provision of critical care beds in England, Wales and Northern Ireland and hence reduced access to critical care for lower risk patients. By contrast, a further study in the USA reported an incidence of 153 per 100,000 population [28] – double that in any other country. This may be due to the significantly higher provision of critical care beds in the USA compared with elsewhere [29], although it may indicate that the ICD codes used to define sepsis in this study were too general, with more specific codes used in later studies [21].

Two previous studies have explored trends in the epidemiology of severe sepsis over a significant time period [19,21]. Both of these were hospital-based studies from the USA, with the diagnosis of severe sepsis based on ICD, Ninth Revision, Clinical Modification (ICD-9-CM) codes. The main results of these studies were consistent with ours, showing increasing population incidence of severe sepsis and decreasing mortality. However, Martin and colleagues [19] also found increasing numbers of organ failures, whereas we found that in a critical care setting the proportions remained constant. Further information on changes over time can be obtained from two similarly designed studies in French critical care units occurring eight years apart [23,30]. The percentage of critical care admissions identified as having severe sepsis increased from 8.4% in 1993 to 14.6% in 2001 – a 74% increase. This was even greater than the 22% increase over 9 years observed in our study. They also found a decrease in mortality from 59% in 1993 to 42% in 2001. These studies may, however, be hindered by the use of very short data collection periods at different times of the year (November to December 1993 and January to February 2001), as severe sepsis in critical care units has been shown to have a strong seasonal pattern with a peak in winter [12]. The trends of an increasing proportion of admissions with severe sepsis and decreasing mortality in this study are also consistent with the earlier analysis of the same database [12].

## Conclusion

The population incidence of critical care admission with severe sepsis during the first 24 hours and associated hospital deaths are increasing in the UK. In 2004, approximately 31,000 adults admitted to general critical care units had severe sepsis within 24 hours of admission and 14,000 of these died before discharge from hospital. These baseline data provide essential information to those wishing to introduce the recently launched Surviving Sepsis Campaign care bundles in UK hospitals. Similar analysis in the future, using data linked to those being collected by the Surviving Sepsis Campaign, may allow the impact of the sepsis care bundles on critical care outcomes to be evaluated.

## Key messages

• 	The number of admissions to UK critical care units with severe sepsis within the first 24 hours of admission is increasing

• 	Mortality for these admissions is decreasing, although the total number of deaths is increasing

• 	In 2004, we estimate there were 31,000 adults admitted to general critical care units in England, Wales and Northern Ireland with severe sepsis

• 	Of these, 14,000 died before discharge from hospital

## Abbreviations

APACHE = Acute Physiology and Chronic Health Evaluation; HDU = high dependency unit; ICD = International Classification of Diseases ICNARC = Intensive Care National Audit & Research Centre; ICU = intensive care unit; PROWESS = Protein C Worldwide Evaluation in Severe Sepsis; SIRS = systemic inflammatory response syndrome.

## Competing interests

The authors declare that they have no competing interests.

## Authors' contributions

DH designed the study, participated in the analysis, and drafted the manuscript. CW participated in the analysis. JE conceived the study. All authors were involved in interpretation of data and critical revision of the manuscript, and have read and approved the final manuscript.

## Figures and Tables

**Table 1:** Admissions with severe sepsis during the first 24 hours following admission to critical care

	Number of critical care units	Unit-years of data	Total number of admissions	Admissions with severe sepsis (%)
1996	61	39.6	14,696	3,458 (23.5)
1997	76	67.4	25,486	6,007 (23.6)
1998	94	85.1	34,033	8,638 (25.4)
1999	103	94.5	38,567	10,257 (26.6)
2000	111	98.6	39,437	10,923 (27.7)
2001	129	110.4	47,688	13,325 (27.9)
2002	148	134.2	59,388	16,285 (27.4)
2003	151	129.3	59,527	16,594 (27.9)
2004	100	54.7	24,905	7,145 (28.7)

Total^a>^	172^b^	814.0	343,860	92,672 (27.0)

^a^Total includes 55 admissions from 1995 and 78 admissions from 2005; ^b^172 critical care units contributed data at any time during the study.

**Table 2:** Changes in components of the SIRS criteria for admissions with severe sepsis during the first 24 hours following admission to critical care, 1996 to 2004

	Highest central temperature^a ^(°C)	Highest heart rate (min^-1^)	Highest non-ventilated respiratory rate^b ^(min^-1^)	Lowest PaCO_2 _(kPa)	Mechanically ventilated, n (%)	Lowest white blood cell count (× 10^9^l^-1^)
1996	38.2 (37.5–38.8)	127 (112–145)	28 (21–36)	4.5 (3.9–5.2)	2,733 (81.0)	11.1 (6.9–15.8)
1997	38.1 (37.5–38.8)	126 (112–144)	28 (22–36)	4.5 (3.9–5.2)	4,744 (79.0)	11.3 (6.9–16.1)
1998	38.1 (37.5–38.7)	125 (110–141)	28 (22–35)	4.5 (3.9–5.3)	6,893 (79.9)	11.3 (7.0–15.9)
1999	38.1 (37.5–38.8)	125 (110–140)	28 (22–36)	4.6 (3.9–5.3)	8,114 (79.1)	11.4 (7.1–16.1)
2000	38.0 (37.4–38.7)	125 (110–140)	28 (22–35)	4.6 (4.0–5.3)	8,624 (79.0)	11.5 (7.1–16.3)
2001	38.0 (37.4–38.7)	124 (110–140)	28 (22–35)	4.6 (4.0–5.4)	10,146 (76.3)	11.8 (7.4–16.6)
2002	38.0 (37.4–38.6)	122 (108–138)	28 (23–36)	4.6 (4.0–5.4)	11,904 (73.7)	11.8 (7.4–16.6)
2003	38.0 (37.4–38.6)	121 (108–138)	29 (23–36)	4.7 (4.0–5.4)	11,986 (73.1)	11.8 (7.3–16.8)
2004	38.0 (37.4–38.7)	120 (107–135)	29 (23–36)	4.6 (4.0–5.4)	5,141 (73.1)	11.9 (7.3–17.0)

Total^c^	38.0 (37.4–38.7)	124 (110–140)	28 (22–36)	4.6 (4.0–5.4)	70,317 (76.4)	11.6 (7.2–16.5)

Values are median (interquartile range) of the most extreme physiological measurement from the first 24 hours following admission to critical care, unless otherwise stated. ^a^Non-central temperature +0.5°C used as a substitute for central temperature in admissions with no central temperature recorded. ^b^For admissions that were not mechanically ventilated for the entire of the first 24 hours following admission to critical care. ^c^Total includes 55 admissions from 1995 and 78 admissions from 2005. PaCO_2_, arterial carbon dioxide tension; SIRS, systemic inflammatory response syndrome.

**Table 3:** Changes in markers of organ dysfunction for admissions with severe sepsis during the first 24 hours following admission to critical care, 1996 to 2004

	Lowest systolic blood pressure (mmHg)	Hourly urine output (ml)	Lowest PaO_2_/FiO_2 _(kPa)	Lowest platelet count (× 10^9^l^-1^)	Lowest pH	Highest base deficit
1996	85 (78–100)	75 (45–114)	20.0 (12.9–28.9)	170 (104–250)	7.28 (7.19–7.35)	5.1 (1.2–9.7)
1997	86 (75–100)	76 (45–114)	19.7 (12.8–28.6)	175 (106–253)	7.28 (7.19–7.35)	5.0 (1.1–9.5)
1998	87 (75–100)	74 (43–109)	19.7 (13.0–28.3)	171 (106–249)	7.28 (7.19–7.35)	4.8 (0.8–9.3)
1999	90 (77–100)	73 (43–109)	19.9 (12.9–28.9)	177 (109–256)	7.28 (7.18–7.35)	4.9 (0.6–9.4)
2000	90 (78–100)	70 (42–106)	19.3 (12.5–28.0)	184 (110–268)	7.28 (7.19–7.36)	4.7 (0.5–9.2)
2001	90 (79–100)	68 (40–103)	20.0 (12.9–28.8)	197 (122–284)	7.28 (7.18–7.35)	4.9 (0.7–9.5)
2002	90 (80–100)	69 (41–104)	19.7 (12.8–29.0)	202 (126–291)	7.28 (7.18–7.35)	4.9 (0.8–9.3)
2003	90 (79–100)	66 (39–101)	19.8 (12.7–28.8)	199 (124–287)	7.28 (7.18–7.35)	5.1 (1.1–9.5)
2004	90 (79–100)	63 (36–98)	19.8 (13.1–29.2)	199 (124–287)	7.27 (7.18–7.35)	4.7 (0.7–9.0)

Total^a^	90 (78–100)	69 (41–105)	19.8 (12.8–28.7)	189 (116–274)	7.27 (7.18–7.35)	4.9 (0.8–9.4)

Values are median (interquartile range) of the most extreme physiological measurement from the first 24 hours following admission to critical care. ^a^Total includes 55 admissions from 1995 and 78 admissions from 2005. FiO_2_, fraction of inspired oxygen; PaO_2_, arterial partial pressure of oxygen.

**Table 4:** Changes in laboratory measurements for admissions with severe sepsis during the first 24 hours following admission to critical care, 1996 to 2004

	Lowest serum sodium (mmoll^-1^)	Lowest serum potassium (mmoll^-1^)	Highest serum creatinine (μmoll^-1^)	Highest serum urea (mmoll^-1^)	Lowest serum albumin (gl^-1^)	Highest serum glucose (mmoll^-1^)	Lowest haemoglobin^a ^ (gdl^-1^)
1996	136 (132–139)	3.7 (3.3–4.1)	121 (84–203)	10.2 (6.3–16.9)	23 (18–29)	9.0 (7.0–12.0)	10.2 (8.8–11.6)
1997	136 (132–139)	3.7 (3.3–4.1)	119 (83–203)	10.1 (6.3–17.1)	22 (17–28)	9.0 (7.0–12.0)	10.0 (8.8–11.5)
1998	136 (133–139)	3.7 (3.3–4.1)	118 (82–197)	9.9 (6.0–16.8)	21 (16–27)	8.9 (7.0–11.9)	10.0 (8.7–11.4)
1999	136 (133–139)	3.7 (3.3–4.1)	115 (80–191)	9.9 (6.0–16.7)	19 (15–25)	8.9 (7.0–11.9)	9.9 (8.6–11.3)
2000	136 (133–140)	3.7 (3.4–4.1)	116 (81–197)	10.0 (6.1–17.1)	19 (14–25)	8.8 (7.0–11.5)	9.7 (8.5–11.1)
2001	137 (133–140)	3.8 (3.4–4.2)	116 (79–195)	10.0 (6.1–16.8)	19 (14–25)	8.6 (6.9–11.3)	9.8 (8.5–11.3)
2002	137 (133–140)	3.8 (3.4–4.2)	113 (80–189)	10.0 (6.0–16.8)	19 (14–25)	8.6 (6.9–11.2)	9.6 (8.3–11.1)
2003	137 (133–140)	3.8 (3.4–4.2)	114 (79–194)	10.0 (6.0–16.8)	19 (14–25)	8.5 (6.8–11.0)	9.5 (8.3–11.0)
2004	137 (133–140)	3.8 (3.4–4.2)	116 (79–194)	9.8 (5.9–16.5)	19 (14–25)	8.5 (6.8–10.8)	9.6 (8.3–11.1)

Total^b^	136 (133–140)	3.8 (3.4–4.2)	116 (80–194)	10.0 (6.1–16.8)	20 (15–25)	8.7 (6.9–11.4)	9.7 (8.5–11.2)

Values are median (interquartile range) of the most extreme physiological measurement from the first 24 hours following admission to critical care. ^a^One-third of haematocrit used as substitute for haemoglobin in admissions with no haemoglobin recorded. ^b^Total includes 55 admissions from 1995 and 78 admissions from 2005.

**Table 5:** Changes in severe comorbities for admissions with severe sepsis during the first 24 hours following admission to critical care, 1996 to 2004

	Immunocompromised^a^	Respiratory comorbidity^b^	Cardiovascular comorbidity^c^	Liver comorbidity^d^	Renal comorbidity^e^
1996	308 (9.1)	231 (7.0)	113 (3.4)	35 (1.1)	60 (1.8)
1997	534 (8.9)	327 (5.6)	161 (2.8)	72 (1.2)	93 (1.6)
1998	714 (8.3)	468 (5.5)	214 (2.5)	134 (1.6)	140 (1.7)
1999	873 (8.5)	480 (4.7)	261 (2.6)	168 (1.7)	172 (1.7)
2000	938 (8.6)	473 (4.4)	243 (2.3)	188 (1.7)	153 (1.4)
2001	1,093 (8.2)	562 (4.2)	262 (2.0)	249 (1.9)	233 (1.8)
2002	1,418 (8.8)	650 (4.1)	323 (2.0)	288 (1.8)	236 (1.5)
2003	1,349 (8.2)	712 (4.4)	292 (1.8)	306 (1.9)	265 (1.6)
2004	615 (8.7)	262 (3.8)	120 (1.7)	104 (1.5)	98 (1.4)

Total^f^	7,849 (8.5)	4,167 (4.6)	1,989 (2.2)	1,544 (1.7)	1,450 (1.6)

Values are number (percentage of admissions with evidence available to assess past medical history) presenting with the specified comorbidity. ^a^Immunocompromised defined as AIDS, daily steroid treatment for past six months, radiotherapy or chemotherapy within past six months, metastatic disease, acute or chronic myelogenous or lymphocytic leukaemia, multiple myeloma, lymphoma, or congenital immunohumoral or cellular immune deficiency state. ^b^Respiratory comorbidity defined as shortness of breath with light activity due to pulmonary disease, or using home ventilation. ^c^Cardiovascular comorbidity defined as fatigue, dyspnoea or angina at rest due to myocardial or peripheral vascular disease (New York Heart Association Functional Class IV). ^d^Liver comorbidity defined as biopsy proven cirrhosis, portal hypertension, or hepatic encephalopathy. ^e^Renal comorbidity defined as requirement for renal replacement therapy for irreversible renal disease. ^f^Total includes 55 admissions from 1995 and 78 admissions from 2005.

## References

[B1] Bone RC, Balk RA, Cerra FB, Dellinger RP, Fein AM, Knaus WA, Schein RM, Sibbald WJ (1992). Definitions for sepsis and organ failure and guidelines for the use of innovative therapies in sepsis. The ACCP/SCCM Consensus Conference Committee. American College of Chest Physicians/Society of Critical Care Medicine. Chest.

[B2] Laterre PF, Levy H, Clermont G, Ball DE, Garg R, Nelson DR, Dhainaut JF, Angus DC (2004). Hospital mortality and resource use in subgroups of the Recombinant Human Activated Protein C Worldwide Evaluation in Severe Sepsis (PROWESS) trial. Crit Care Med.

[B3] Abraham E, Wunderink R, Silverman H, Perl TM, Nasraway S, Levy H, Bone R, Wenzel RP, Balk R, Allred R (1995). Efficacy and safety of monoclonal antibody to human tumor necrosis factor alpha in patients with sepsis syndrome. A randomized, controlled, double-blind, multicenter clinical trial. TNF-alpha MAb Sepsis Study Group. JAMA.

[B4] Abraham E, Laterre PF, Garbino J, Pingleton S, Butler T, Dugernier T, Margolis B, Kudsk K, Zimmerli W, Anderson P (2001). Lenercept (p55 tumor necrosis factor receptor fusion protein) in severe sepsis and early septic shock: a randomized, double-blind, placebo-controlled, multicenter phase III trial with 1,342 patients. Crit Care Med.

[B5] Bernard GR, Vincent JL, Laterre PF, LaRosa SP, Dhainaut JF, Lopez-Rodriguez A, Steingrub JS, Garber GE, Helterbrand JD, Ely EW (2001). Efficacy and safety of recombinant human activated protein C for severe sepsis. N Engl J Med.

[B6] Ziegler EJ, Fisher CJ, Sprung CL, Straube RC, Sadoff JC, Foulke GE, Wortel CH, Fink MP, Dellinger RP, Teng NN (1991). Treatment of gram-negative bacteremia and septic shock with HA-1A human monoclonal antibody against endotoxin. A randomized, double-blind, placebo-controlled trial. The HA-1A Sepsis Study Group. N Engl J Med.

[B7] Rivers E, Nguyen B, Havstad S, Ressler J, Muzzin A, Knoblich B, Peterson E, Tomlanovich M (2001). Early goal-directed therapy in the treatment of severe sepsis and septic shock. N Engl J Med.

[B8] Dellinger RP, Carlet JM, Masur H, Gerlach H, Calandra T, Cohen J, Gea-Banacloche J, Keh D, Marshall JC, Parker MM (2004). Surviving Sepsis Campaign guidelines for management of severe sepsis and septic shock. Crit Care Med.

[B9] Levy MM, Pronovost PJ, Dellinger RP, Townsend S, Resar RK, Clemmer TP, Ramsay G (2004). Sepsis change bundles: converting guidelines into meaningful change in behavior and clinical outcome. Crit Care Med.

[B10] Linde-Zwirble WT, Angus DC (2004). Severe sepsis epidemiology: sampling, selection, and society. Crit Care.

[B11] Padkin A, Rowan K, Black N (2001). Using high quality clinical databases to complement the results of randomised controlled trials: the case of recombinant human activated protein C. BMJ.

[B12] Padkin A, Goldfrad C, Brady AR, Young D, Black N, Rowan K (2003). Epidemiology of severe sepsis occurring in the first 24 hrs in intensive care units in England, Wales, and Northern Ireland. Crit Care Med.

[B13] Harrison DA, Brady AR, Rowan K (2004). Case mix, outcome and length of stay for admissions to adult, general critical care units in England, Wales and Northern Ireland: the Intensive Care National Audit & Research Centre Case Mix Programme Database. Crit Care.

[B14] Knaus WA, Draper EA, Wagner DP, Zimmerman JE (1985). APACHE II: a severity of disease classification system. Crit Care Med.

[B15] Harrell FE (2001). Robust estimation of the covariance matrix. Regression Modeling Strategies: With Applications to Linear Models, Logistic Regression, and Survival Analysis.

[B16] CMA Medical Data (2001). Directory of Critical Care.

[B17] Efron B, Tibshirani RJ (1993). An Introduction to the Bootstrap.

[B18] National Statistics Online. http://www.statistics.gov.uk/.

[B19] Martin GS, Mannino DM, Eaton S, Moss M (2003). The epidemiology of sepsis in the United States from 1979 through 2000. N Engl J Med.

[B20] Flaatten H (2004). Epidemiology of sepsis in Norway in 1999. Crit Care.

[B21] Dombrovskiy VY, Martin AA, Sunderram J, Paz HL (2005). Facing the challenge: decreasing case fatality rates in severe sepsis despite increasing hospitalizations. Crit Care Med.

[B22] Zahorec R, Firment J, Strakova J, Mikula J, Malik P, Novak I, Zeman J, Chlebo P (2005). Epidemiology of severe sepsis in intensive care units in the Slovak Republic. Infection.

[B23] Brun-Buisson C, Doyon F, Carlet J, Dellamonica P, Gouin F, Lepoutre A, Mercier JC, Offenstadt G, Regnier B (1995). Incidence, risk factors, and outcome of severe sepsis and septic shock in adults. A multicenter prospective study in intensive care units. French ICU Group for Severe Sepsis. JAMA.

[B24] Alberti C, Brun-Buisson C, Burchardi H, Martin C, Goodman S, Artigas A, Sicignano A, Palazzo M, Moreno R, Boulme R (2002). Epidemiology of sepsis and infection in ICU patients from an international multicentre cohort study. Intensive Care Med.

[B25] Silva E, Pedro MA, Sogayar AC, Mohovic T, Silva CL, Janiszewski M, Cal RG, de Sousa EF, Abe TP, de Andrade J (2004). Brazilian Sepsis Epidemiological Study (BASES study). Crit Care.

[B26] van Gestel A, Bakker J, Veraart CP, van Hout BA (2004). Prevalence and incidence of severe sepsis in Dutch intensive care units. Crit Care.

[B27] Finfer S, Bellomo R, Lipman J, French C, Dobb G, Myburgh J (2004). Adult-population incidence of severe sepsis in Australian and New Zealand intensive care units. Intensive Care Med.

[B28] Angus DC, Linde-Zwirble WT, Lidicker J, Clermont G, Carcillo J, Pinsky MR (2001). Epidemiology of severe sepsis in the United States: analysis of incidence, outcome, and associated costs of care. Crit Care Med.

[B29] Angus DC, Sirio CA, Clermont G, Bion J (1997). International comparisons of critical care outcome and resource consumption. Crit Care Clin.

[B30] Brun-Buisson C, Meshaka P, Pinton P, Vallet B (2004). EPISEPSIS: a reappraisal of the epidemiology and outcome of severe sepsis in French intensive care units. Intensive Care Med.

[B31] Participating units [ICNARC – Intensive Care National Audit & Research Centre]. http://www.icnarc.org/audit/cmp/participating-units/.

